# Mechanomyographic Parameter Extraction Methods: An Appraisal for Clinical Applications

**DOI:** 10.3390/s141222940

**Published:** 2014-12-03

**Authors:** Morufu Olusola Ibitoye, Nur Azah Hamzaid, Jorge M. Zuniga, Nazirah Hasnan, Ahmad Khairi Abdul Wahab

**Affiliations:** 1 Department of Biomedical Engineering, Faculty of Engineering, University of Malaya, Kuala Lumpur 50603, Malaysia; E-Mails: marufibitoye@yahoo.com (M.O.I.); khairi@um.edu.my (A.K.A.W.); 2 Department of Biomedical Engineering, Faculty of Engineering and Technology, University of Ilorin, Ilorin, P.M.B. 1515, Nigeria; 3 Department of Exercise Science, Creighton University, 2500 California Plaza, Kiewit Fitness Center 228, Omaha, NE 68178, USA; E-Mail: JorgeZunigaUlloa@creighton.edu; 4 Department of Rehabilitation Medicine, Faculty of Medicine, University of Malaya, Kuala Lumpur 50603, Malaysia; E-Mail: nazirah@um.edu.my

**Keywords:** mechanomyographic signal, MMG parameters, muscle performance, isokinetic, isometric, electromyogram

## Abstract

The research conducted in the last three decades has collectively demonstrated that the skeletal muscle performance can be alternatively assessed by mechanomyographic signal (MMG) parameters. Indices of muscle performance, not limited to force, power, work, endurance and the related physiological processes underlying muscle activities during contraction have been evaluated in the light of the signal features. As a non-stationary signal that reflects several distinctive patterns of muscle actions, the illustrations obtained from the literature support the reliability of MMG in the analysis of muscles under voluntary and stimulus evoked contractions. An appraisal of the standard practice including the measurement theories of the methods used to extract parameters of the signal is vital to the application of the signal during experimental and clinical practices, especially in areas where electromyograms are contraindicated or have limited application. As we highlight the underpinning technical guidelines and domains where each method is well-suited, the limitations of the methods are also presented to position the state of the art in MMG parameters extraction, thus providing the theoretical framework for improvement on the current practices to widen the opportunity for new insights and discoveries. Since the signal modality has not been widely deployed due partly to the limited information extractable from the signals when compared with other classical techniques used to assess muscle performance, this survey is particularly relevant to the projected future of MMG applications in the realm of musculoskeletal assessments and in the real time detection of muscle activity.

## 1. Introduction

A number of research outputs have identified the significance of mechanomyographic signal (MMG) parameters in the skeletal muscle research field. MMG represents the mechanical manifestation of the neurophysiological phenomena underlying muscle contraction and the mechanical counterpart of the electrical activity of the motor units [1]. Motor units (MU) represent the basic functional block of the neuromuscular system [2,3]. Several research attempts have demonstrated the correlation of MMG amplitude to the motor unit recruitment and used MMG frequency features to extract information about the global firing rate of the unfused activated MU [4]. Therefore, simultaneous analysis of the temporal and spectral features of the mechanomyographic signal have been consistently useful in describing the differences in motor control strategies that modulate force generation during isometric and dynamic muscle activities in voluntary [5] and non-voluntary muscle activities [6]. In addition, during muscle contractions, changes in the muscle fiber geometry, indirectly recorded as mechanomyographic signals reflect the slow bulk movement of the muscle (*i.e.*, lateral oscillations generated by the muscle at its own resonance frequency [7,8], and the pressure waves due to the muscle fibre dimensional changes [9]). This fact supports the application of MMG signal parameters as indicators of MU activation patterns and firing rates in contracting muscles [10,11]. Consequently, MU activation and firing rates depend on the contraction intensity of muscles [12,13]. Thus, the application of MMG to monitor muscle function during fatiguing and non-fatiguing contraction to quantify the voluntary and evoked muscle performances can no longer be considered trivial as the analysis of the signal in time and frequency domains involves complex signal processing procedures [14].

MMG is useful in various areas of interest because its characteristic parameters can be delineated in the time domain including the root mean square (RMS), peak to peak (PTP) amplitude and mean average value (MAV), and in the frequency domain which includes the mean power frequency (MPF), median frequency (MDF), center frequency (CF), and frequency variance (FV). Recently, the MMG parameters have been characterised and classified in the joint time-frequency domain including time scale variables, wavelets, and energy distributions among other representations. These features provide valuable information about the contractile properties of skeletal muscles' motor units used to estimate the force capacity and fatigability [15]. Measurements derived from the MMG power spectrum have served as essential features to discern the underpinning factors responsible for various physiological changes during different muscle activities. In effect, MMG may serve as a reliable estimation of the skeletal muscle force and its derivatives (*i.e.*, muscle torque and power) used as indices of the muscle performance with implications in prosthetics design [16], orthopedics and rehabilitation sciences [17], the assessment of work related musculoskeletal disorders [18], and in diagnosis of neuromuscular disorders [19]. MMG offers a non-invasive means to assess muscle intrinsic functions which can be optimised if an appropriate signal processing technique is deployed to extract the signal features.

As a growing area of research, investigators have conducted comprehensive reviews on the feature extraction methods of MMG. Beck *et al.* [20] undertook a survey to describe the relationship between MMG signal parameters and dynamic muscle action. They highlighted that valid neuromuscular information on the muscle action could be derived from MMG features. Alves and Chau [21] substantiated the application of MMG-wavelet transform coefficients as an efficient index to detect muscle activities. Recently, the application of the wavelet-based feature analysis of MMG signals has been introduced to delineate postural control strategy used to study the effect of muscle fatigue on the mechanical properties of the skeletal muscle which may not be evident in ordinary wavelet transform (WT) coefficients of the signal [22]. In essence, a number of studies have demonstrated the validity and reliability of MMG signal variables in the analysis of skeletal muscle activities [17,23].

However, despite the ease of collection due to the sensing flexibility and the validated significance of the signal parameters to estimate various aspects of skeletal muscle activities, to the best of our knowledge, there has not been any documented effort that has presented a comprehensive point of view on the classical and modern techniques used to extract MMG parameters with highlights on the clinical applications. Thus, the objective of this study was to conduct an appraisal of the effectiveness of common MMG features' extraction methods, their recent derivatives and other representations during skeletal muscle activities with implications in clinical practices. This review focuses specifically on the methods used to extract the temporal, spectral, and the joint time frequency parameters of MMG signals underlying the static and dynamic skeletal muscle during voluntary and non-voluntary activities. However, the review does not relate, except for a brief note, to more comprehensive methods and technical exploratory information as could be found in the previous reviews [4,24–27] dedicated to those purposes. This attempt investigated topical issues related to the strengths and limitations of the available MMG features' extraction methods in view to highlight the reliable standard methods to widen the clinical applicability of the signal modality.

## 2. Technical Characteristics of MMG Sensors, Selection Criteria and Signal Acquisition

The considerable variation of the transducing technology in the available MMG sensors has been validated [28]. However for the purpose of standardization and signal reproducibility, the characteristics of the sensors used for MMG signal acquisition are suggested to be consistent in terms of the outcomes to be measured, though the signal shape may vary based on the physical quantity measured (e.g., accelerometer measures acceleration and laser displacement sensor measures displacement) [29]. During the skeletal muscle contraction, the surface MMG signal may be acquired on the surface of the skin in the form of acceleration, originally termed ‘acceleromyogram’, sound termed ‘phonomyogram or acoustic myogram’ [30], displacement and vibration termed ‘vibromyogram’ [31]. However, by way of consensus, all these measurements have been collectively called mechanomyogram (MMG). The type and size of the muscle and the nature of the muscle action are important factors considered in sensor selection for a particular study.

Typically, the characteristics of a reliable MMG transducer include the following: (a) high sensitivity in the muscle vibrational frequency range, *i.e.*, 2 Hz up to 100 Hz and low sensitivity to random signals (noise); (b) ease and standardization of the sensor attachment; (c) biocompatibility and applicability in a clinical environment; and (e) cost effectiveness when compared with other clinical assessment techniques [32] to mention the major considerations. Once the reliability and physical characteristics of a sensor is ascertained and there is no physical interference with the muscle surface dynamics, including the light weight (5–40 g) consideration, such a sensor could readily serve as a candidate for the signal acquisition [33].

Further, to obtain MMG signals with acceptable integrity, the established technical guidelines used for electromyograms (EMGs) are often adopted [34]. The uniformity of the distance between the intended signal site and the sensor is crucial. It has been demonstrated that different results may be obtained between trials if distance varies [35]. There are evidences to affirm that more signal could be collected over the muscle belly than the fascia at the muscle border or towards the tendon [8,36] corroborating the significant relationship between the magnitude of the signal and the relative distance of the sensor from the muscle belly. However, there is an isolated report on the high level of association between sensors' placement in different axis/plane on the same muscle [37] that led the investigators to suggest that the shape of MMG signal waveform may not be sensitive to the sensor placement and motor unit but the amplitude of the signal does [38,39]. Meanwhile, for very large muscles including pectoralis major, trapezius or latissimus dorsi, MMG recordings from the same site may not represent the whole muscle mechanical action as both the shape of waveforms and the amplitude will be sensitive to the sensor placement [37].

Another consideration during MMG acquisition is ensuring the sensor firmness to the skin surface and contact pressure standardization to reduce the variability of the signal during repeated recordings [35]. The consistency of the signal response also depends significantly on the uniformity of the sensor location between trials. Bolton and co-workers [40] demonstrated that the predominant frequency (below 20 Hz) of the power spectrum of MMG signal is known to correlate positively with the sensor firmness to the skin surface. Further, the skin fold thickness may affect the MMG recordings because of the low pass filtering effect of the tissue between the target muscle and the detection sensor [41]. Efforts to reduce the effects of noise and artifacts may necessitate the skin preparation before affixing the sensor. However, MMG signals are not influenced by changes in the skin impedance, and thus, may not require rigorous skin preparation during acquisition [42]. Nevertheless, for an improved signal integrity, the standard practice include the use of double sided adhesive tape to fix the MMG's sensor to the skin in order to ensure a constant pressure. Isolation of irrelevant muscles through an experimental setup on standard testing devices such as custom made or commercially available dynamometers has been suggested to limit the yet unavoidable effects of cross-talk [43].

In experimental designs where more than one subject are recruited, investigators may seek a comparison between muscles, tasks and/or individuals, thus, a normalization of the acquired signal is recommended. There is inter individual variability that may adversely influence the signal including differences in muscle mass, muscle length and the thickness of the tissue between the sensor and the muscle. To eliminate the effect of the muscle variations, normalization to a reference level is often prescribed.

Normalization constitutes a means of adjusting data to conform to a common scale for an objective averaging and analysis in order to validly compare between muscles, task and individuals [44]. Normalization is equally important because the MMG signal is variable and dependent on many factors (including subcutaneous tissue, intramuscular fluid pressure, muscle stiffness and muscle temperature) that are practically impossible to control and each one of these factors varies between one subject to the other. Normalization facilitates comparison between electrode sites on the same muscle, two different muscles, and between days [45]. Therefore, normalization is recommended to prevent erroneous conclusions especially if the test is meant to compare between trials, between electrode re-applications, between different muscles and different candidates [46]. However, Zuniga and co-researchers verified that normalization may not be sufficient to eliminate the influence of inconsistent sensors placement between trials and thus suggested the importance of standardized electrode placement especially for comparison purposes [47]. Equally, if the MMG measurements could be obtained in the fundamental units of vibration from the skeletal muscles, *i.e.*, m/s^2^ for the contraction (since sensors' sensitivity is often presented in mV/g where g is the acceleration due to gravity when accelerometer sensor is used) the need to normalize may be unnecessary [6]. This procedure equally affects MMG signals measured with other physical sensors including the condenser microphone and piezoelectric contact sensor (measured in volts) only that the gravity consideration is excluded and the sensor sensitivity factor should also be considered (*i.e.*, to match/convert the voltage level to the physical measurements). In any case, a meticulous attention to sensor placements and sensor selection based on the purpose of acquisition and the type of muscle action should be a priority.

Since MMG signals are inherently mechanical, the signal acquisition task may be facilitated because the signal can be collected without the need for a separate circuitry to eliminate the electrical noise interfaces especially 50 Hz noise. Equally, the signal acquisition may be realised with a single uniaxial electrode configuration unlike the simplest monopolar configuration of EMG (often unused) with a separate reference electrode whereas bipolar and multipolar configuration of EMG require even more electrodes. These facts collectively inform the few hardware requirements [48] in MMG acquisition which in effect, may result in the cost effectiveness of MMG signal processing. However, there is consensus regarding the adverse effect of the motion artifact and interference on MMG signal especially during dynamic contractions. Tremor, clonus and other forms of artifacts are supposedly inherent characteristics of contraction that generate the muscular mechanical vibration [49]. These interferences have been reported with frequency below 10 Hz and thus may overlap with the frequency spectrum of MMG which has its main frequency below 100 Hz [31]. Therefore, the removal of these artifacts is crucial to obtaining a reliable MMG signal [50].

Current efforts to mitigate the effect of motion artifacts include: the application of Fourier truncation [51], the use of coherence analysis [52], the experimental precaution to exclude unwanted voluntary movement [53], the use of standard muscle isolation equipment such as dynamometers (*i.e.*, the retraining straps on the dynamometers isolate only the relevant muscle), and through high pass filtering [54]. The cut-off frequency of the filter is flexible based on the type of sensor, muscle and muscle action, but usually within 5–100 Hz in most reported studies on human subjects. A unique approach of an application of a coupled microphone-accelerometer sensor without depending on the filtering manipulation was suggested by Silva and Chau [55]. Although their design demonstrated a marked distinction between the motion artifact and the useful MMG signal, the effectiveness of the generalization and thus, external validity of the design on able bodies and individuals with functional disabilities has not been substantiated. Therefore, none of the available solutions is without its technical limitations. Consequently, there has not been a comprehensive understanding and solution to mitigate the adverse effect of motion artifact in the literature [56]. The majority of researchers have, therefore, recommended the use of the sensor that is less affected by the motion artifact including condenser microphones [29] and piezoelectric contact sensors [57], especially during muscles' dynamic contractions and areas where limb movements are unavoidable [58].

Theoretically, methodological and physiological concerns may dictate the signal sampling rate based on the site of the muscle of interest. A common compromise is to sacrifice the storage space for high sampling rate during the signal acquisition exercise. The rule of thumb based on the Nyquist-Shannon sampling theorem is that; “for a reliable reproducibility and representation, a signal should be sampled at least twice the highest frequency content of the signal” [59]. This suggests that MMG should be sampled at least 200 Hz since the highest recorded MMG signal frequency is below 100 Hz [4]. However, the common sampling rate as found in the literature is 1000 Hz (1000 samples/s) or 2000 Hz [60], presumably to check the aliasing effect which may be due to the hardware limitations. Oversampling enables a “sufficient accuracy of the MMG signals' cross-correlation time measurements to detect a delay corresponding to the fastest transverse vibration in the muscular medium” as described by Ouamer *et al.* [61]. This approach has been used to demonstrate that MMG signal reflects the muscle response as a global resonant structure to the local fluctuations of pressure during voluntary contractions [61]. However, if the concern is to select the highest possible sampling rate, there is usually a point at which sampling above a certain threshold have no additional information—“a point of diminishing return”. In order to save system storage, it can be safely proposed that the sampling frequency may be kept within the standard limits.

Collectively, as the reliability of a recorded MMG signal is influenced by the relative size of the sensor and the firmness to the skin surface, so are locations and orientation of the sensor on the muscle, the thickness of the subcutaneous fat layer, and the noise and artifact reduction technique adopted. Reliable MMG signals require an appropriate selection of signal analysis methods [20]. Equally pertinent is that a proper guide should be given to the candidates in order to perform the experimental test/trial identically with each repetition through training and familiarization with the experimental protocol and equipment.

## 3. MMG Parameters' Extraction Methods

Applications of MMG signal parameters in muscle research for experimental decisions have been well documented. Diverse methods of time domain and frequency domain marked the beginning of the investigation into the signal analysis. Different types of joint time-frequency domain analysis were proposed lately to extract MMG parameters because of the non-stationarity of the signal. Selection of an appropriate signal processing technique is crucial for the objectivity of the analysis of MMG signals obtained during muscle activities. During an isometric contraction, where the signal may be assumed to be stationary, the application of the traditional parameters extraction methods is widely accepted. However, in a dynamic muscle action, changes in muscle length, number of active motor units, and the thickness of the tissue between the muscle and MMG sensors affect the MMG amplitude and frequency, resulting in non-stationary signals [8,41,62]. Thus the application of the classical time series analysis methods may no longer be valid.

In order to characterize the signal responses, the time domain's PTP, RMS, MAV or average rectified values (ARV), and the frequency domain's MDF and MPF [63] and the FV are the common variables often explored in MMG analysis. However, due to some useful information that may not be ordinarily evident in time or frequency domain, various algorithms have been suggested for the joint time-frequency domain representations of the signal including short time Fourier transform (STFT), wavelet transform (WT) and the relatively recently suggested time scale representations. These modalities have been widely used to estimate the muscle contractile information embedded in the signal. However, lack of consensus concerning the consistency in the methods used may suggest that investigators have relied on the selection of the algorithm depending on the type of muscle actions and the purpose of the parameter extraction.

The renewed interest in MMG signal relies on the use of the well-established EMG theory and signal processing techniques to investigate MMG signal characteristics [34]. Therefore, the theoretical background of EMG signal have been logically applied to relate MMG signals to the underlying neurophysiological phenomena of the muscle during contraction since the two signal modalities measure skeletal muscle's contractile properties. Subsequent to the MMG signal acquisition, signal cleaning, parameter extraction followed by classification are necessary in order to determine if the signal parameter significantly retains contractile information usable for the reliable estimation of muscular activities. These have been used to support the real time practical implementations and to determine the consistency of the reliability of the signal modality to assess muscle performance over time [53]. Applications of MMG signals features had offered useful insights into describing motor unit activation strategies [64], muscle force assessment [65], muscle fatigue monitoring [66], discrimination of muscle fiber typing [67], control portable neuromuscular training system in order to evaluate the residual muscle capability in individuals with limited voluntary skills [68] and clinical examination of neuromuscular disorders. MMG has equally been used as biopotential and control signals in the neuromuscular electrically evoked contractions and in muscle machine interfaces [23,69] to mention a few.

**Figure 1.**
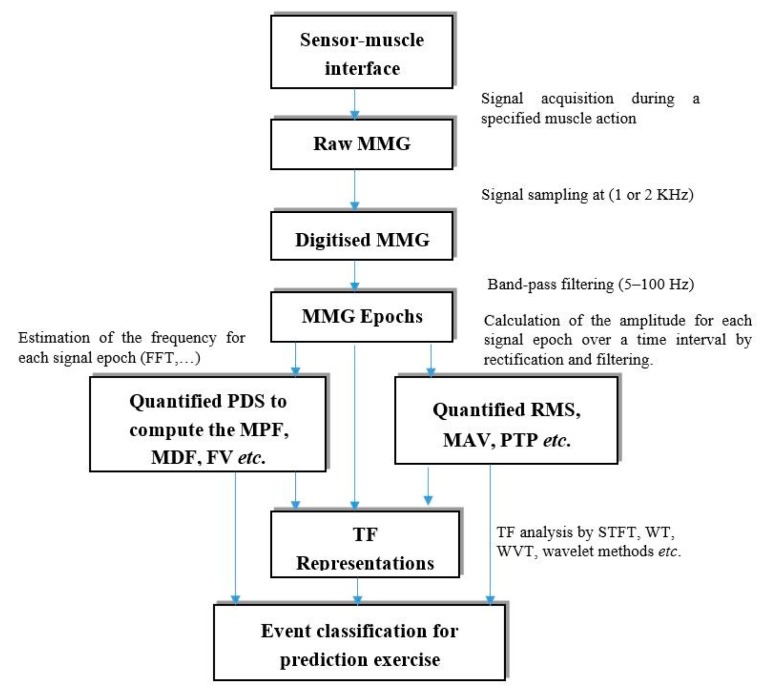
Basic steps in MMG parameters extraction.

The traditional approach to MMG signal power spectra computation is Fourier based transforms used to convert from the time domain to the frequency domain or *vice versa*. However, the complex relationships between the time and frequency (TF) content of the signal have been examined by using techniques such as WT, STFT, and Wigner-Ville Distribution (WD) to compute the TF representations. While most recently, various linear wavelet analysis algorithms were proposed in muscle research with reliable classification performance, the need for continuous reassessment of these methods is warranted [20] as the information extractible from the linear MMG signal analysis methods is not sufficient to describe fully the physiological phenomenon underlying the generation of the MMG signal during different tasks [70]. Although we may not have exhausted all available citations, the overview of the relevant literature on the MMG parameter's extraction methods were adequately represented as summarized in Figure 1.

### 3.1. Time Domain Analysis

#### MMG Amplitude Estimation

**Figure 2.**
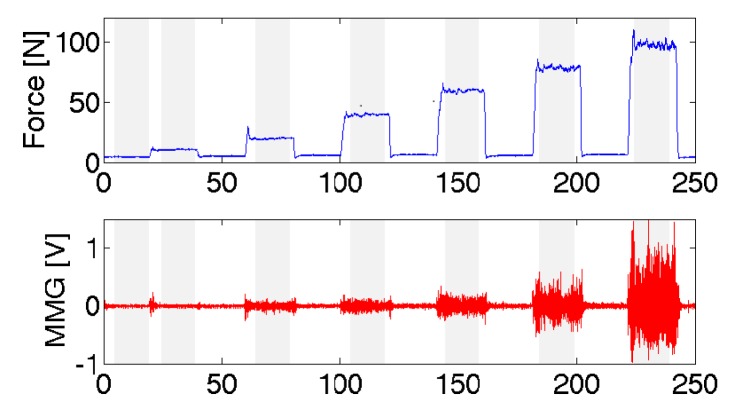
An example of an increase in the magnitude of MMG amplitude with increasing isometric force levels (5, 10, 20, 40, 60, 80, 100) N for 20 s contraction and 20 s resting between contractions [78].

There are several time domain processing techniques used to extract information using MMG amplitude. The estimation of the muscle effort, monitoring of muscle fatigue and the examination of neuromuscular disorders have been delineated by the changes in the time domain features of the MMG signal which generally signifies changes in motor unit recruitment during muscle contractions [18,71–76]. Before the estimation of RMS amplitude information of MMG signal, the acquired (raw) time series signal is rectified, smoothed and band-pass filtered at around (5 Hz–100 Hz) [77] by a zero phase 4th order Butterworth filter depending on the site of acquisition and commonly sampled at 1000 Hz or 2000 Hz. The RMS amplitude, as a measure of the magnitude of the varying value is the square root of the mean square value defined for a specific time interval, T in seconds. An important objective of this exercise is to obtain indices of muscle force [65]. The amplitude of the signal depends on the muscle fiber fluctuations under tension [29], increases with increasing muscle force as a result of the high contraction level as shown in Figure 2 [78], and provide information on the level of muscle activation that may be required for functional tasks [20]. The RMS feature of MMG may therefore correlate with muscle effort [79] and has been used to estimate the muscle torque [80]. Akataki and colleagues demonstrated an estimation of motor unit activation strategy underlying voluntary force generation by the use of MMG-RMS [81]. These indicate the significant relationship between the muscle force and MMG-RMS and suggest why RMS is considered the most reliable parameter in the time domain [82]. However, care must be taken in relating the MMG-RMS to the muscle effort as the association between the two variables varies based on the muscle architecture and dimension (thickness, cross-sectional area and volume), motor unit composition and the pattern of contractions that may influence the muscle fiber fluctuations under tension on which the muscle performance depends [83]. Therefore, researchers have suggested the muscle-specific differences in the response of MMG amplitude [20].

Other common representations of MMG amplitude features are the mean amplitude value (MAV) or average rectified value (ARV) which is the average of the absolute value of the MMG signal over a specific time interval T in seconds, *i.e.*, the time windowed mean of the absolute value of the signal. The ARV is characterised with a lower coefficient of variation when compared with RMS during MMG analysis [84]. Further, the peak to peak (PTP) distance is one of the metrics of a signal amplitude representing the distance between the signal peak (highest amplitude value) and the trough (lowest amplitude value). Although not usually used for time series amplitude representation, but the PTP amplitude has been adopted by a few isolated studies where the variable was applied to monitor changes in mechanical properties of the muscle during fatigue in voluntary [85] and electrically evoked contractions [86]. The consistent decrease or the lack of recovery in the value of PTP amplitude of MMG has been used to indicate the reduction of muscle force during the period of stretching [87], sustained contraction or muscle fatigue [23,85]. This phenomenon has been ascribed to the persistent changes in the viscoelastic characteristics of the series elastic component manifested as the reduction in the force generating capacity of the muscle [87].

The commonly used MMG amplitude representations can therefore, be defined by the following Equation (1) through Equation (3):

(1) $$\begin{array}{ll} \text{RMS}=\sqrt{\frac{1}{N}\sum_{i=1}^{N}x_{i}^{2}} & \text{where}\sum_{i=1}^{N}x_{i}^{2}=(x_{1}^{2}+x_{2}^{2}+x_{3}^{2}+\ldots +x_{N}^{2}) \end{array}$$

(2) $$\begin{array}{ll} \text{MAV}=\frac{1}{N}\sum_{i=1}^{N}\left|x_{i}\right| & \text{where}\sum_{i=1}^{N}\left|x_{i}|=(x_{1}+x_{2}+x_{3}+\cdots +x_{N}\right) \end{array}$$

(3) $$\text{PTP}=2\sqrt{2}\text{RMS}$$

where, *x_i_* represents the *i* th sample of the MMG signal and N is the number of samples in the segment considered.

Accordingly, the assessment of the level of muscle contraction can be obtained by the time series analysis of MMG signal [80]. However, because of the tendency of the signal to be contaminated by muscle tremors and other forms of mechanical artifacts, the accurate prediction of the muscle torque and fatigue often demands further analysis.

### 3.2. Frequency Domain Analysis

There are diagnostic and assessment features of MMG signals that are more evident in the frequency domain. Different patterns in the spectral analysis during muscle contraction have been reported. The common experimental practice includes the use of a shift in the frequency feature of MMG to track muscle fatigue. The magnitude of the changes is dependent on the type of muscle action, *i.e.*, dynamic (eccentric or concentric) or static (maximal or submaximal effort). The measure of the magnitude and pattern of the spectral compression has been demonstrated by the frequency features of the signal with significant clinical implication [88]. The mean frequency is the most widely used frequency feature of the MMG signal compared to the median and the peak frequencies because it is less affected by the method of analysis [89] and is an important metric used to examine muscle mechanical changes underlying the muscle activation [90]. Generally, the frequency content of the MMG signal reflects changes in the global firing rate of an unfused activated motor units during muscle contraction [91].

#### 3.2.1. Fourier Based Methods

##### Fast Fourier Transform (FFT)

Practical signals can be delineated in frequency domain by Fourier transform representation. In order to investigate the frequency domain behavior, the frequency component of MMG must be characterized. The standard practice involves the application of the fast Fourier transform algorithm to compute the power density spectrum of MMG [36], from which the frequency features of MMG signal could be derived. FFT is the gold standard used to convert time or space to frequency or *vice versa* by projecting the data set in a space of sine and cosine. The algorithm has been described as the most important numerical algorithm and characterises with a high computational efficiency [92]. To obtain a reliable spectrum, it is vital that the time series signal should be noise free. However, since MMG signal is a biological signal, the artifact is always subdued by the use of band-pass filters.

Because not all the features of signals are evident in time domain, investigators have always estimated the frequency content of the signal based on the recommendation of Diemont *et al.* [90] and as computed by Kwatny *et al.* [93] to estimate the MPF (the average of frequencies in the power spectrum). Equation (4) represents the MPF.

(4) $$\text{MPF}=\frac{\int_{0}^{fs/2}f.S(f)df}{\int_{0}^{fs/2}S(f)df}$$

where *S (f)* and *fs* are the power density spectrum (PDS) of the signal and the sampling frequency respectively. MPF represents the moment of the PDS and the major indicator of spectra changes:

(5) $$\text{MDF}=\int_{0}^{\text{MDF}}S(f)df$$

(6) $$=\int_{\text{MDF}}^{\infty }S(f)df$$

(7) $$=\frac{1}{2}\int_{0}^{\infty }S(f)df$$

The MMG signal's MDF, though not commonly used due to the reported relatively low reliability [94], it is however, the frequency that splits the power spectrum of MMG into two equal halves [95]. The Equations (5)–(7) represent MDF:

(8) $$\text{FV}=\frac{\int_{0}^{\text{fs}/2}{\left(\text{f}-\text{MPF}\right)}^{2}\text{S}(\text{f})\text{df}}{\int_{0}^{\text{fs}/2}\text{S}(\text{f})\text{df}}$$

The frequency variance (FV) shown in Equation (8) [80] measures the scale or degree of the signal spread in a power spectrum density. As a significant feature of the MMG signal, FV shows a marked decrease during incremental voluntary contraction. The FV represents an MMG feature suitable for torque estimation when the signal is interfered by noise [80]. Investigators have demonstrated that the aforementioned frequency domain parameters showed variations between different muscle actions and between normal muscles and those with neurological disorders. These facts seem especially relevant in muscle research because it can be used to discriminate and identify motor unit control strategies underlying different muscle actions [96] and the firing rates [64].

Equally, as the foremost frequency domain estimator with pronounced limitations during non-static contraction due to changes in the motor unit recruitment and firing rates and the underlying muscle length changes, the trend of recent studies have continued to uphold the suitability of the FFT algorithm to compute the frequency parameters of MMG signal in isometric muscle contractions [62]. Essentially, transforming to frequency domain allows access to more information that could not be evident in the time series representation only. Although, the frequency spectrum generated by the FFT algorithm displays the magnitude and phase of the time domain data [97], investigators have verified that the algorithm may not be suited for signals with transient components.

The FFT algorithms assume the stationarity of an input signal and may not be suitable to analyse dynamic muscle actions. There are other classical model based techniques which have not been widely used including maximum entropy spectrum estimation [4,90,98] and autocorrelation technique [12]. The probable reason for the low application of these classical algorithms may be due to the surge of advanced and robust signal processing techniques and the consequent fast and intelligent processing. The general drawback of frequency domain analysis is the inability to track the rapid changes in the frequency content of the input signal [20].

### 3.3. Time Frequency (TF) Representations

During the analysis of dynamic contractions of muscles, there is need to rapidly track the frequency changes (in time) during certain muscle activities that reflect the motor unit recruitment and firing rate to estimate the torque generation across the range of motion considered [99]. However, changes in the muscle fiber length, thickness of the tissue between the muscle and MMG sensor, and the number of active motor units and their firing rates are responsible for the non-stationarity of the underlying MMG signal [100]. In effect, the signal possesses more information beyond what can be entirely evident in the analysis of the time domain and its power spectrum. In such a situation, the limitation of the Fourier based method regarding the assumption of signal stationarity fails, thus, the inability of the technique to estimate features embedded in the joint TF domain is evident because of the missing time scale information. Accordingly, the joint TF signal processing techniques including the wavelet transform (WT), short-time Fourier transform (STFT), Wigner-Ville transform, have been suggested to analyse the signal during these tasks because of their robustness to reflect rapid changing muscles' mechanical properties underlying different muscle actions [11,81].

The major compromise associated with a high TF resolution is the considerable amount of time required for computation [101]. Nevertheless, the joint TF analysis methods have significantly improved our knowledge of estimation of muscle action when the frequency content of the MMG signal is rapidly changing with time. The immediate current usage of these methods is in the understanding of motor control strategies that could be used to study torque modulation which has lent significant impetus to the study and estimation of muscle fatigue. The commonly used TF analysis techniques as reported in the literature are described in the following sections.

#### 3.3.1. Short time Fourier Transform (STFT)

The short time Fourier transform theoretically involves the breaking down of the waveform into a number of short segments [102]. The analysis of each resulting segment of signal is conducted by the classical FT techniques. The equation representing STFT for signal *x*(*t*) is as shown in Equation (9) [103]:

(9) $$X(t,f)=\int_{-\infty }^{\infty }x(\tau )w(t-\tau )e^{-j\pi ft}d\tau$$

where *w*(*t* − *τ*) represents the window function, τ variable slides the window across the waveform, *x*(*t*).

A spectrogram can be obtained from the square magnitude of the STFT. Thus the energy distribution of the signal along the frequency direction at a given time can be computed [104]. However, the inherent limitation of the STFT is the trade-off between the time and frequency resolution [81]. Notably, the application of STFT was used by Akataki and colleagues to calculate the RMS and MPF during ramp contraction of the first dorsal interosseous muscle [105]. This is perhaps because the condition of the MMG signals' non-stationarity was proposed and observed. Equally, in a related application, STFT was demonstrated with marked MMG-TF changes across the force spectrum during the analysis of isometric ramp contraction [99]. Essentially, the TF representation by STFT is a valid algorithm used to track the changes (*i.e*., in time) in MMG signal frequency with force development during muscle contractions [99]. However, the STFT requires the selection of a predefined time window which may lead to a compromise of frequency resolution because the algorithm is unable to generate an appropriate time resolution for distinguishing high-frequency events along with adequate frequency resolution for distinguishing low frequency components [88,102].

#### 3.3.2. Wavelet Transform (WT)

Unlike Fourier based methods, wavelet methods do not assume signal stationarity and project signals into orthogonal space function called wavelets (*i.e.*, functions that oscillate for a short period, localized in time and frequency and their integral over time must be zero). The wavelet transform can be used to examine changes in the spectral density of a signal using an enhanced frequency resolution derived from wavelets. The wavelet transform reflects discontinuity better than FT and is deemed more suited to decompose signal to its fundamental form and reveals transient characteristics of muscle action which may not be evident in Fourier transform algorithms [106]. Since the WT examines frequency changes over time, it may be particularly useful in filtering and noise reduction while preserving the information content of the signal. Extraction of TF feature of MMG signal by WT has been used to predict signal qualities from the signal's dataset. The general equation of WT is as shown in Equation (10):

(10) $$W(a,b)=\int_{-\infty }^{\infty }x(t)\frac{1}{\sqrt{|a}|}\psi^{*}\left(\frac{t-b}{a}\right)dt$$

where *x*(*t*) is the input signal, *ψ* is the probing function, and *a* is the variable that varies the time scale of the probing function, while *b* acts to translate the function across the input signal *x*(*t*).

The wavelet transform algorithm has proven more appropriate than the Fourier transform approach especially when dealing with non-stationary signals, though with more intensive computation [104]. This has been illustrated in several applications including the examination of the pattern of MMG responses to the dynamic muscle action of biceps brachii [107], the assessment of muscle force and examination of the changes in the spectrum of MMG when muscles fatigue [76]. Therefore, with the proper selection of wavelet function and scaling factor, it may be possible to achieve a reliable discrimination and classification of muscle actions during different tasks and interventions. In most cases, both STFT and WT usually give fairly identical pattern when force changes during fatiguing contraction as reflected in the MMG signal spectrum [99]. Unlike in STFT, the frequency resolution is not compromised and there is a general relationship between the time scale and frequency component (time-frequency resolution) [88]. Consequently, reflection of changes in the WT may continue to play useful roles in the real time analysis of muscle activities especially in clinical population [21]. However, the wavelet transform is not an orthogonal transformation, thus, the distribution of the power among different wavelets require special consideration [24]. This may serve as a limitation in some applications.

#### 3.3.3. Wigner Ville Transform (WVT) Methods

The Wigner Ville transform (WVT) method adopts the TF energy distribution method to estimate the power spectral function of a non-stationary signal [108]. As a special case of Cohen's class, WVT generates a simple, time dependent spectra representation of a signal. The WVT of a real signal is represented by the following Equation (11), with *x*(*t*) representing the signal of interest [109]:

(11) $$W\left(t,w\right)=\int_{-\infty }^{\infty }x\left(t+\frac{\tau }{2}\right)x^{*}\left(t-\frac{\tau }{2}\right)e^{-jwt}d\tau$$

WVT algorithms are suited to resolve the events in the test signal of MMG both in time and frequency domain and particularly useful in expressing frequency information of the signal. The WVT has been used to analyse the MMG in TF domain to illustrate that the MMG signal could reflect the muscle resonance frequency during contraction [11]. However, the WVT algorithm often generates interference terms that dominate the events distribution, thus reduce the signal ability to identify the primary signal terms [110,111]. The algorithm is equally not suited for the analysis of multicomponent signals [111]. The existence of noise limits the application of WVT and smoothed version/derivatives and may not be appropriate to represent signal with high signal to noise ratio considerations [112].

#### 3.3.4. Recent Wavelet Analysis Methods

These are mainly the time scaled methods used to extract both the time and frequency information from a signal with superior capacity to separate simultaneous events in time and frequency. The necessity to obtain the timing information as frequency of the signal changes is typical of various applications and analysis of biopotentials. The method of wavelet analysis has several advantages over the traditional TF analysis methods. This is because the method retains a relatively constant relationship between the time and frequency resolution across the entire frequency range of the MMG signal without interference. One of the variations of the recent wavelet analysis method was proposed by von Tscharner [24] for intensity analysis of the TF features of surface myoelectric signal but later adopted to analyse MMG signal by Beck and colleagues [110]. The approach describes the power intensity of the MMG signal as a function of both time and frequency and may be achieved by repeating the wavelet transform for all wavelets [24].

Specifically, MMG wavelet analysis has been shown to provide distinctively the intensity, timing and the frequency of the muscular events embedded in the time frequency domain of MMG signal. The algorithm has been used to show a distinct spectral band for concentric and eccentric isokinetic action of the quadriceps muscle group. The method demonstrates the capability of MMG amplitude to identify the muscle specific differences in each wavelet band during isometric force level in the superficial quadriceps muscles [113]. The modality has also been used to assess the postural control and the effect of fatigue on the mechanical properties of the skeletal muscle using MMG signal [22]. The practical application of the method is as described by Beck *et al*. [113].

The wavelet analysis method monitors the functional aspects of muscle activation since it is event and intensity oriented [24] and may be used to illustrate the muscle spectral band during activation. In general, the wavelet analysis is well suited for analysing the non-stationary MMG signal including dynamic contraction and enhances the flexibility of TF representations [114]. The algorithm computes spectra in a very short time window and in a short time interval. It equally gives an overview of the spectral, temporal, and intensity attributes of the signal during different types of muscle actions [110]. The MMG wavelet analysis retains a fairly constant relationship between time and frequency resolution across the entire frequency range of MMG (approximately 2–120 Hz). However, the MMG wavelet analysis is unable to reliably examine chirp-like signals (signals that are not band limited) because the algorithm performed well only within the frequency range of 5–100 Hz [110].

Further, the effectiveness of the wavelet packet methods was recently validated by Xie and colleagues where the decomposed MMG signal features were used as a multifunction prosthesis control with a high average classification accuracy of 89.7% [115]. Equally, Al-Mulla and Sepulveda, demonstrated the application of a wavelet analysis method to classify (with accuracy of 80.63%) the fatigue content in MMG signal from the biceps brachii muscles in healthy volunteers [116].

#### 3.3.5. Other TF Analysis Methods

##### Hilbert-Huang Transform (HHT)

As a relatively new time-frequency analysis method, the Hilbert-Huang transform (HHT) was initially proposed in the field of fluid mechanics [117], but has gained popularity in the analysis of potentials of biological origin [118]. For flexibility and ease of the spectral parameter estimation especially in non-linear and non-stationary biopotentials, HHT is described with empirical mode decomposition and Hilbert spectral analysis has been proposed [119]. The HHT algorithm was introduced to generate a reliable assessment of muscle fatigue using the spectral parameters of electromyographic signal. Although the algorithm has not been substantiated to analyse MMG signals, the cue from other TF methods that were used to analysis both EMG and MMG [32] suggests that application of HHT in MMG analysis may be timely considering its ability to improve the estimate of MPF [120], and assessment of muscle fatigue as demonstrated by Peng and colleagues [121].

Collectively, there are a number of important considerations in the selection of methods for MMG signal analysis. These factors may include; the nature of contraction (stationary or dynamic), the information to be extracted (embedded in time (T), frequency (F) and/or joint TF domain representations), the frequency band of the information content of the signal, and perhaps the speed of computation and the precision of representation. Table 1 summarizes the common MMG signal features extraction methods. The classification of the strength and weakness of the methods highlighted in the table was based on the observations and suggestions made in the previous investigations [11,24,79,99,110,111].

## 4. Clinical Applications of MMG Parameters in the Analysis of Muscle Contraction Activities

### 4.1. MMG Signal Parameters and Muscle Force

**Figure 3.**
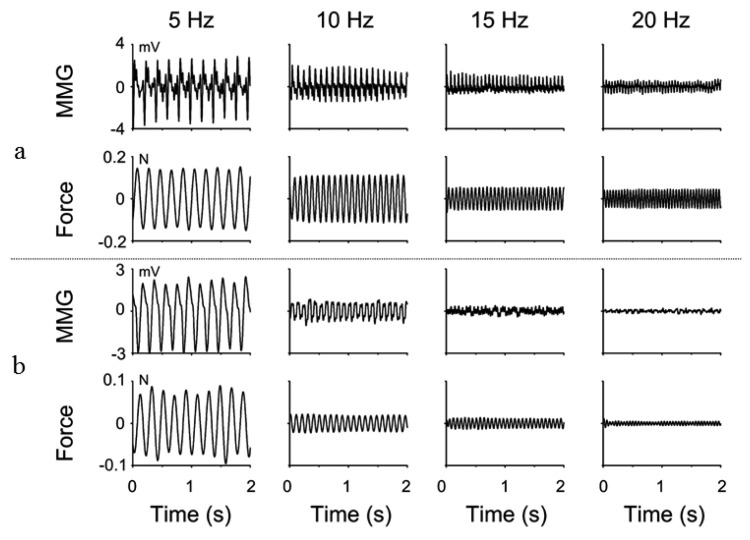
Relationship between MMG amplitude and force during repetitive electrical stimulation (5 Hz, 10 Hz, 15 Hz, 20 Hz) of a motor unit (MU) (**a**) MU with shortest twitch duration (163.2 ms) (**b**) MU with longest twitch (220.6 ms) from the media gastrocnemius muscle of a healthy volunteer. Systematic reductions in the MMG amplitude and force reductions were evident as the stimulation frequency increased [128].

In rehabilitation and exercise sciences where clinicians and exercise physiologists may wish to prevent injury and improve performance, the direct measurement of the muscle force may not be feasible [122] and the conventional approach of estimating muscle force with EMG potentials has been described with limitations [123–126]. During a sustained muscle action the EMG response increases with increasing fibre recruitment, despite a decreasing muscle force [127], suggesting that this signal modality may not be suitable in this domain. The relationship between the selected feature of MMG signal and the muscle force signifies an interesting index of the muscle performance. This relationship is evident in different muscles during different types of muscle actions and activations. The amplitude of MMG signal is correlated with the level of muscle effort and can be used to discriminate levels of muscle activation during voluntary and evoked contractions (Figure 3). This association is consistently reported linear up to 80% maximum voluntary contraction during isometric muscle action [128]. Therefore, the experimental and clinical practices are influenced since most voluntary muscle activities are within the submaximal level of muscle effort.

The estimation of muscle force during dynamic muscle action is more relevant during the assessment of skeletal muscle functional activities [36]. There are indications of a linear relationship between the amplitude of MMG signal and the muscle force during concentric and eccentric contractions [20]. However, the muscle force generated during concentric contraction has been generally reported higher than eccentric contraction as often evident by the amplitude and MPF of MMG due to the differences in MU recruitment strategies. Ebersole *et al.* [76], demonstrated the unique contribution of superficial muscles of quadriceps femoris to the control of generated muscle force during the repeated, concentric muscle actions by MMG amplitude and MPF. The sensitivity of MMG signal to follow muscle activities at different force levels [17] including constant force, declining force, increasing force and during fatiguing contraction is due to the ability of the signal to monitor the firing rates and recruitment strategies of the motor units.

Motor unit recruitment and firing rates are the major mechanisms on which the production of muscle force depends. These mechanisms have been used to assess muscle force at different levels of contractions [129]. Therefore, the consistencies in the relationship between MMG-RMS and force/torque production provide evidence on the close relationship between the motor unit control strategies (firing rates and recruitment) [105]. However, the validity of this evidence has not been sufficiently substantiated as there are few models [130,131] and on limited muscles to evaluate the significance of this relationship. The limitation of this signal modality in muscle research, therefore, could not be entirely ascertained based on available literature, further investigation is however necessary in order to guide clinical decisions based on MMG signal parameters.

Another approach to muscle force assessment is through isokinetic dynamometry (ID). The ID technique represents a convenient and an objective means to study the force-velocity characteristic of muscle groups [132,133]. The ID informs how the assessment of muscle groups control the loading imposed on them. In isokinetic testing, the resistance imposed on the subject by the device is equal to the subject's effort. Thus, the subject resistance is equal to the effort applied. Theoretically, an isokinetic dynamometer measures the generated muscle force by controlling the velocity of the movement and measuring the force applied through an arrangement of strain gauges (force transducers) [134]. Apart from being costly and a lack of portability, isokinetic dynamometry requires certain level of expertise before usage [17] and may not be used for self-assessment.

The current trends indicate that the pattern of MMG frequency parameters during isometric and isokinetic voluntary contraction may discriminate the task and be muscle specific [113]. For instance, the force contribution of each muscle in the superficial quadriceps femoris muscle group could be discriminated by the MMG wavelet band despite the fact that the rectus femoris (RF), vastus lateralis (VL) and vastus medialis (VM) are all innervated by a common nerve, the femoral nerve. However, other muscles including hamstrings, gastrocnemius and soleus, which are thought to be responsible for maintaining posture and required for exercise with clinical implication in rehabilitation and injury prevention are yet to be investigated. Nevertheless, there appears to be a consensus on the pattern of MMG frequency response and the muscle force. Alteration in the pattern of time and frequency domain parameters of MMG are indicative of the muscle force during contraction activities [51]. MMG may represent a valid and reliable alternative to force signal when the latter is difficult to assess [135]. Investigators continue to propose other MMG indices including the robust Multistate Lempel-Ziv (MLV) index to assess MMG-force relationship [65]. Given that few investigators have examined the MMG response as an index of muscle force to the level of adequate clinical acceptability, future investigations to further understand factors which may influence the amplitude and frequency features of MMG signal to broaden its clinical relevance are clearly warranted.

### 4.2. MMG Signal Parameters and Electrically Evoked Contraction Activities

**Figure 4.**
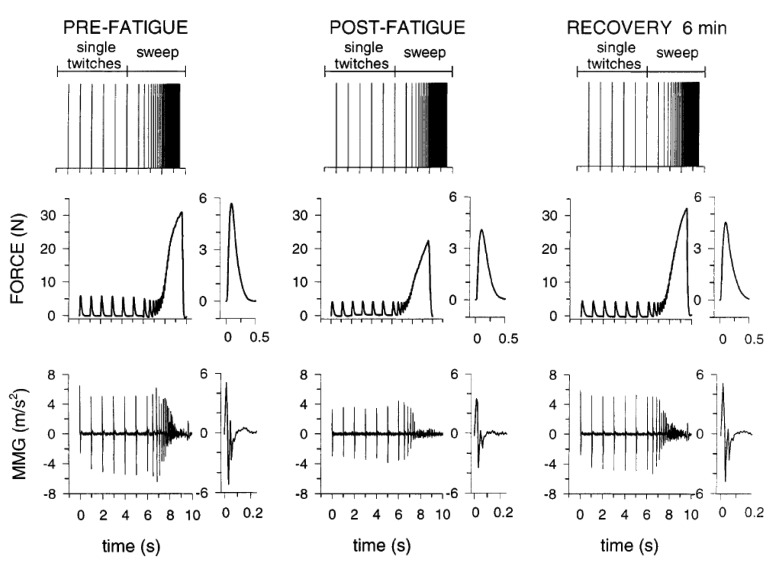
The relationship between the stimuli evoked human tibialis anterior muscle's mechanical changes during pre fatigue, post fatigue, recovery state and MMG-PTP amplitude. Stimulation pattern (six single twitches and the 1–50 Hz sweep), the force, and MMG responses from top down respectively. A pattern of relation is visibly evident (redrawn from Orizio *et al.*, [85]).

The characteristics of MMG parameters do not only depend on the muscle being studied, but are also related to the muscles' state of health (normal or paretic), stiffness, temperature and the depth of the subcutaneous tissue [136]. There are indications of viable relationships between electrical stimulus evoked mechanical muscle changes during fresh, fatiguing, post fatigue, recovery state and MMG-PTP amplitude (Figure 4) suggesting the legitimacy of MMG magnitude to estimate muscle performance from a specific muscle group [85]. However, variations in individuals' physiological composition, muscle length, level of lesion- for individuals with neurological conditions and the inherent rapid fatigue are among factors that may influence the efficacy of the neuromuscular electrical stimulation (NMES) induced contraction especially in spinal cord injured (SCI) population [137].

An efficient NMES rehabilitation can be achieved by a stimulation protocol that optimizes muscle force [138] to enhance restoration of the lost muscle function and promotes health. Theoretically, the property of neuroplasticity that influences the metabolic and contractile properties of the denervated muscle is exploited in effective NMES applications [139]. To date, NMES has been clinically evaluated and described with limited clinical impact. There is consensus [140] on the factors responsible for this observation including the very technical setup of the NMES, the inability of the device to manage the rapid occurrence of muscle fatigue in the target population (SCI) and inefficient control of activated muscles. Inconsistent muscle assessment methods that may be applied as a modulating signal in closed looped NMES devices may also be responsible. Attempts have been made on the application of some biopotentials (*i.e.*, as modulating signals) including EMG and MMG to mention the most common [141]. During electrically evoked contraction [142] especially in a less trained muscle where more current is required to recruit the muscle fibers [112], there is an implication of an increase in the stimulation artifact which has a confounding effect on the integrity of the EMG signal. Equally, the issue of activation-contraction delay especially when relating EMG signal to the mechanical output measurements (*i.e.*, force, torque, joint angle, *etc.*) [143] and the consensus on the dissociation of muscle force and EMG during fatiguing contraction clearly indicate the limitation of EMG as a modulating or control signal [36].

Alternatively, the application of MMG signal as a NMES modulating biopotential in electrically stimulated muscles has been recently proposed and has since received some clinical attentions [23]. Impetus from the suggestion of Barry and colleagues (*i.e.*, on the application of MMG as a control signal for an externally powered prosthesis) [69] as advanced and validated by other researchers [21,115,144] that verified the application of MMG potentials as a control signal could well be further applied in NMES protocols. The insight gathered from their research outputs and suggestions was applied by Alves and Chau [145] to demonstrate the signal capability as a control signal in the rehabilitation of disabled individuals in order to optimise the rehabilitation outcomes.

Currently, the most effective stimulation parameter is yet to be agreed upon. The reason for this inconsistency may be due to the lack of consensus regarding “what constitutes the appropriate stimulation parameters” because the stimulation patterns available are not equally effective in different candidates with varying degree of lesions. If the muscle activation pattern can be reliably discerned by MMG signals there may be a justification for its application as an outstanding biopotential NMES modulating signal as proposed by Gobbo and colleagues [23], since the signal modality is immune to electrical artifacts and it is noninvasive.

## 5. Discussion

We have presented various methods used to extract MMG parameters and different studies confirming the wide application of those parameters to examine various aspects of muscle activities with experimental and clinical applications. The limitations of each technique have been enumerated aiming at limiting the erroneous interpretation and conflicting report often encountered in the literature. A number of domains where MMG parameters have been demonstrated to describe the basic muscle activation pattern and areas of potential application were highlighted herein.

Contrary to the traditional signal modality used as the control signal in human machine interfaces, including EMG, which is described as an index of superficial muscle activities [146] and characterised with high sensitivity to the skin impedance, among other constraints, the literature has shown that MMG could offer a reliable and viable alternative option. This is based on the fact that MMG propagates through the soft tissue, possesses entirely different frequency range of interest [29], could be recorded with different types of sensors signifying the flexibility of its sensing technology and its sensors are immune to skin impedances and cheaper to fabricate [144]. Therefore, MMG signals may be a powerful metric of muscular neurophysiological functions once the proper signal processing is performed.

Within the limitations of the studies investigated, the wavelet analysis method a time scaled method used to estimate the joint time frequency features of MMG is significantly more robust in terms of its applicability in the real time muscle activity classification. This fact lends more support to the joint TF approach in the analysis of stochastic and non-stationary MMG signals as previously suggested by Alves and Chau [21]. However, time series analysis may suffice to assess the level of muscle activation. In situations where the investigator aims to estimate how MMG signal reflects changes in the global firing rate of an unfused activated motor units during MU recruitment, the analysis of the frequency content of MMG may be sufficient. Clearly, indices of the muscle performance, not limited to force, power, work, fatigue [147] could be estimated from MMG parameters.

Understanding the pattern of muscular events embedded in MMG features during voluntary and stimulus evoked contraction would better inform the design of exercise and rehabilitation to prevent injuries, enhance performance and promote healthy living. This will facilitate flexibility in the treatment modalities and provides enormous support to the projected therapeutic and functional outcome of NMES rehabilitation intervention in disabled individuals. As a signal modality that is rich in the information on the state of neuromuscular systems, MMG signal features have been well explored to examine various aspects of skeletal muscle activities. However, there are still wide knowledge gaps in the sensor technology to manage the muscle tremor. Attempts to reduce the gap include integration of two different sensors to improve sensing performance and signal integrity [55]. Issues requiring further investigation include limiting the effect of factors such as; muscle temperature, stiffness, mass, sensor location in the muscle [148], intramuscular pressure, tension, the viscosity of the intracellular and extracellular fluid mediums, and the firing rates of the active motor units on MMG magnitude [4].

It has been established that MMG signals are related to MU activation rate and the muscle fiber contractile properties (*i.e.*, contraction and relaxation). However, the direct relationship between the MUs activation strategy and the MMG signal's parameters remains debatable [64]. Therefore, researchers have continued to rely on the indirect association of MMG parameters and motor units control strategy [129]. Having identified some factors (including intramuscular fluid pressure, muscle stiffness, muscle temperature) that could impair the MMG signal parameters, efforts to clarify the direct pattern of relationship between the MU and MMG signal is therefore, warranted.

### Suggestions for Further Studies

MMG signal modality has limitations in terms of its scope of application. The current efforts on the sensor development and advanced signal processing techniques are expected to continually widen the application of the signal in the experimental and clinical practices. To broaden the realm of MMG applications, the adaptation of the EMG signal processing techniques to analyse MMG signal may not always be reliable [115]. Therefore, we envision that the reassessment of the presented methods and exploration of new ones (especially non-linear techniques) may help to elucidate the mechanism underlying muscle activities and facilitate the real time muscle activity detection.

There is lack of consensus regarding the sensor placement and orientation. Some investigators suggest a meticulous consideration to sensor placement due to the effect of spatial distribution on the relationship between MMG features and muscle force irrespective of the muscle size [63] while some argued that the sensitivity of the signal waveform to the different sensor orientation is limited only to very large muscles [39]. Efforts to clarify this phenomenon are necessary in setting the standard for sensor placement irrespective of the muscle size.

There is need to consistently examine the reliability of signals if their clinical application is envisaged [149]. The reliability of the classical signal modality including EMG has been described with clear limitations that has continued to guide its application in various fields of relevance. This is due to extensive investigation of the signal in its areas of potential applications. Therefore, MMG signal reliability requires consistent evaluation in their various domains of applications. Different methods of MMG parameters extraction in healthy candidates have been described. While some investigators have proposed new algorithms as aforementioned, the accuracy of those methods may not always be valid and reliable for analysis of muscles in individuals with neurological disability because of the marked differences in their muscle physiology (*i.e.*, deficient MU activation). Further experimental validation and clinical assessment of the available parameters extraction methods in such population may be informative.

Although the MMG signal parameters have been fairly applied to quantify muscle performance, it is pertinent for the investigators considering its incorporation into clinical assessment to be aware of the strength and limitation of each standard technique described to forestall erroneous estimations and interpretations. The unification of the patterns highlighted will give a common framework nurtured by researchers' consensus. Avoidance of factors that may affect the reliability of the signal is equally of immense importance. Our knowledge of the mechanical behavior of the muscle signal during activation has been improved [74]. In some instances, identified limitations of the signal are partly due to the inherent nature of the signal and may not be entirely dependent on the type of extraction method adopted. Information about the site of the signal may inform the potential artifact to contend with.

## 6. Conclusions

An appraisal of the standard practice in MMG signal parameters extraction with implications in clinical practice has been conducted. The recent advances in the applications of the signal features as control signals in muscle machine interfaces, as practical proxies of muscle force and the proposition of the signal as a modulating signal in NMES devices (because the signal is insensitive to the stimulation artifact) bespeak the signal relevance in muscle research. Notwithstanding the fact it represents a non-invasive signal to assess the muscle performance, a marked deficiency in the available methods to mitigate the tremor artifact in MMG is equally evident in the literature. This query remains a valid research question for which the signal modality may continue to be subjected to challenges mitigating against its projected potentials in muscle research and rehabilitation. Nevertheless, the current level of research outputs serves as impetus for the development of robust and reliable muscle MMG linear and non-linear models. The need for rehabilitation researchers to consistently consider the nature of the signal before deploying the parameter extraction methods has been highlighted. It is hoped that this review will be a useful point of reference for future studies in the application of MMG signal. Collectively, more clinical evaluations of muscles during different tasks are encouraged, supported by further development of computational tools and researches into new algorithms for muscle performance analysis and their validations using MMG signal. The results of this review are relevant to experimental and clinical domains where MMG parameters may be of immense use.

## Figures and Tables

**Table 1.** Summary of the common MMG signal features extraction methods.

**Method**		**Strength**	**Weakness**	**Area of Applications**	**Reference**
**Type**	**Domain**				
R&F	Time	Simple analysis procedure.	Sensitive to environmental interfaces, muscle tremor and deformation.	Assessment of muscle force to estimate the muscle contraction level/effort. Assesses the relative strength of the signal and contraction.	[11]
FFT	Frequency	Analysis with the application of the frequency shift of the power spectrum.	Inability to track the rapid changes in the frequency content of the input signal.	Estimates the level of muscle contraction/effort. Patterns of the spectral compression have been widely used to track muscle fatigue.	[79]
STFT	TF	Simply tracks spectral variation with time.	It requires the selection of a predefined time window which may lead to a compromise of the frequency resolution.	Signal decomposition and classification. Assessment of force changes especially during ramp contraction.	[99]
WT	TF	Multi-resolution representation, good frequency and time resolution.	The distribution of the power among different wavelets require special consideration.	Signal decomposition and classification. Automation of the muscle fatigue estimation	[111]
WVT	TF	The Power spectrum displays good localization properties. Energy conservation (energy of the signal can be easily obtained).	It generates interference terms (noise that overlaps the signal terms, and disallows the high TF resolution) that dominate the events distribution. Not suited to analyse multi component signal.	Identification of the muscle resonance frequency during contraction.	[11,110]
Wavelet analysis	Time scale	Computes spectra in a very short time window and interval. Gives an overview of the spectral, temporal, and intensity attributes of the signal.	Inability to analyse chirp-like signal, *i.e.*, signal that are not band limited.	Signal decomposition and classification. Reliable estimation of the muscle fatigue phenomenon. Well suited to analysis non-stationary signals.	[24,110]

Abbreviation: R&F: Rectification and filtering; FFT: Fast Fourier transform; STFT: Short time Fourier transform; WT: Wavelet transform; WVT: Wigner Ville transform; TF: Time frequency.
